# Persistence of Unintegrated HIV DNA Associates With Ongoing NK Cell Activation and CD34+DNAM-1brightCXCR4+ Precursor Turnover in Vertically Infected Patients Despite Successful Antiretroviral Treatment

**DOI:** 10.3389/fimmu.2022.847816

**Published:** 2022-04-26

**Authors:** Lucia Taramasso, Federica Bozzano, Anna Casabianca, Chiara Orlandi, Francesca Bovis, Sara Mora, Mauro Giacomini, Lorenzo Moretta, Mauro Magnani, Antonio Di Biagio, Andrea De Maria

**Affiliations:** ^1^ Infectious Diseases Clinic, IRCCS Policlinico San Martino Hospital, Genoa, Italy; ^2^ Department of Biomolecular Sciences, University of Urbino Carlo Bo, Urbino, Italy; ^3^ Biostatistics Unit, Department of Health Sciences, University of Genoa, Genoa, Italy; ^4^ Department of Informatics, Bioengineering, Robotics and System Engineering (DIBRIS), University of Genoa, Genoa, Italy; ^5^ Immunology Research Area, Bambino Gesù Children's Hospital IRCCS, Rome, Italy; ^6^ Department of Health Sciences, University of Genoa, Genoa, Italy

**Keywords:** HIV, reservoir, natural killer, residual replication, inflammatory precursor, CD34, ART, unintegrated HIV-DNA

## Abstract

The quantification of proviral DNA is raising interest in view of clinical management and functional HIV eradication. Measures of all unintegrated HIV DNA (uDNA) forms in infected reservoir cells provides information on recent replication events that is not found from other proviral DNA assays. To evaluate its actual relevance in a cohort of perinatally-infected adult HIV patients (PHIV), we studied how peripheral blood mononuclear cell uDNA levels correlated with total HIV DNA (tDNA) and with overall replication or innate immune control parameters including NK cell activation/exhaustion and lymphoid turnover. Twenty-two PHIV were included, with successfully controlled HIV (HIV RNA <50 copies/mL) on combined antiretroviral therapy for mean of 8.7 ± 3.9 years. uDNA accounted for 16 [5.2-83.5] copies/µg and was strongly correlated with tDNA (ρ=0.700, p=0.001). Flow cytometric analysis of peripheral NK cells showed that CD69 expression was directly correlated uDNA (p=0.0412), but not with tDNA. Interestingly, CD56^-^CD16^+^NK cells which include newly described inflammatory precursors and terminally differentiated cells were directly correlated with uDNA levels (p<0.001), but not with tDNA, and an inverse association was observed between the proportion of NKG2D^+^ NK cells and uDNA (ρ=-0.548, p=0.015). In addition, CD34^+^DNAM-1^bright^CXCR4^+^ inflammatory precursor frequency correlated directly with uDNA levels (ρ=0.579, p=0.0075). The frequencies of CD56^-^CD16^+^ and CD34^+^DNAM-1^bright^CXCR4^+^ cells maintained association with uDNA levels in a multivariable analysis (p=0.045 and p=0.168, respectively). Thus, control of HIV-1 reservoir in aviremic patients on ART is an active process associated with continuous NK cell intervention and turnover, even after many years of treatment. Quantification of linear and circular uDNA provides relevant information on the requirement for ongoing innate immune control in addition to ART, on recent replication history and may help stratify patients for functional HIV eradication protocols with targeted options.

## Introduction

A hallmark of lentivirus infection is the persistence of integrated or partly episomal DNA in long-lived cells—referred to as the reservoir—which guarantees lifelong infection (1). Accordingly, infection with human immunodeficiency virus type 1 (HIV-1) determines the establishment of the viral DNA reservoir of long-lived CD4+ cells (2). Indeed, lentiviral reservoirs are established very early in acute infections (3) and maintain replication competence even during antiretroviral treatment (ART), leading to a lifelong need for medication (4). The characterization of HIV reservoirs during ART over time and strategies for elimination or control represent a major scientific focus to achieve HIV eradication (5–7).

HIV DNA in CD4+ PBMC is a measure of total reservoir size (1, 5, 6) and rapidly declines after ART initiation, but it remains static afterward, tending to plateau after the first years of ART (8) and persisting even after 4-12 years of successful ART (9, 10). Within integrated nuclear HIV DNA sequences, partially modified or deleted HIV sequences may lead to attenuated disease in exceptional instances (11). About 3% of HIV reservoir sequences are fully replication competent in patients requiring ART to control HIV replication (12) and is in line with prompt viremic rebound on ART interruption (13) independent of more potent newer regimens (14). Low level viremic persistence indeed occurs in many patients on ART with suppressed plasma HIV RNA levels <50 copies/mL for a prolonged time, and a low level of viremia can still be detected by ultrasensitive assays (15, 16).

A fraction of HIV DNA is unintegrated (uDNA) and assays measuring these extrachromocosomal forms have been used to monitor ongoing intra-cellular viral replication events. Indeed, uDNA HIV in both linear and circular form represents a short-lived preintegration phase of HIV-1 latency and is considered a consequence of a residual viral replication in ART-treated patients (17, 18) Accordingly, uDNA assays are not used to estimate the viral reservoir because the majority of uDNA may degrade and contributes little to viral production (19).

Several studies showed that uDNA can still be measured during successful ART using an improved assay, even when plasma viremia is below the cut-off for common clinical tests, thus signaling recent replication events (17, 20, 21).

Reservoir persistence is long-lived and has been attributed to the inherent resistance of HIV reservoirs to HIV-specific CD8+ CTL (7, 22, 23). Indeed, *in vitro* suppression by CD8+ T cells of viral reservoir activation is largely independent of cytotoxic CD8+ T lymphocytes and relies on C-C chemokines and other soluble factors (24). Opposite to their potency in controlling virus replication CD8+ CTLs are apparently inefficient in controlling HIV reservoir. There are so far few or minimal indications that during HIV latency there is relevant antigen presentation to CD8+ CTLs. In addition using potent CD8+CTLs together with latency reversing agents and quantitative viral outgrowth assays, results in the elimination of a subset of CD4+ T cells cells harboring defective HIV proviruses, but not of those harboring infectious proviruses thus suggesting a relative inefficiency of CD8+CTLs in controlling viral reservoir (22). On the other hand, Natural Killer cells (NK) are instrumental in the control of HIV replication under specific conditions, such as in Elite- or HIV-controller patients where, in the absence of any antiretroviral treatment they associate with improved levels of HIV reservoir size and control (25–28). In this regard, NK cells appear to significantly contribute to shaping and containing the HIV-1 reservoir since their functional activity in both Elite Controller and ART-related suppressed patients inversely correlates with patients’ HIV reservoirs both *in vivo* and *in vitro* (29). Accordingly, their analysis could provide useful insights in the shaping of HIV reservoir under controlled conditions.

In perinatally HIV-infected children (PHIV) born to infected mothers, early ART shapes the HIV reservoir and the immune response (30–32). PHIV patients who were born in the early 90s and are now adolescents or young adults did not have the opportunity for early ART and were sometimes exposed to months or years of suboptimal antiretroviral regimens prior to combined antiretroviral therapy (cART) (33, 34). In this context, viremia copy years (VCY), a metric of cumulative HIV RNA burden calculated based on longitudinal viral load data, has been used to summarize in a single value the viral burden of years (35, 36). VCY is considered a reliable marker of past viral burden and has been associated with mortality and organ damage in people living with HIV and PHIV (36, 37).

Low-level viremic persistence in suppressed ART-treated patients contributes to sustain the reservoir size and may occur upon standard antigen-specific responses to daily non-HIV related antigenic stimulation of latently infected CD4+ T cells [e.g.: vaccination, transient infections (38, 39)]. These events occur in tissues where drug concentrations may be insufficient to fully suppress replication (40) thus leaving space for different immune control in different patients, and may be revealed by uDNA. These considerations together with the role played by Natural Killer (NK) cells in HIV reservoir control in Elite Controllers (29), raised the question whether circulating NK-cell phenotype and NK cell turnover could reveal their involvement in underlying HIV low-level replicative events in ART-treated patients. To this end we performed a combined evaluation of HIV DNA reservoirs and of VCY in a PHIV cohort that has survived two decades of infection with the aim of describing their relationship with NK cell activation/exhaustion markers and lymphoid turnover.

## Materials and Methods

### Study Cohort

Our center has been involved from the beginning of the HIV pandemic in the follow-up and description of the natural history of mother-to-child transmission (41). For this reason, we have continuously cared for patients surviving HIV infection since the early years when ART was unavailable. We here present data on the cohort of patients originally enrolled in the European Collaborative Study (42) who survived and were not lost to follow-up over the years.

The study was performed in accordance with the ethical standards laid down in the 1964 Declaration of Helsinki and its later amendments and in accordance with Italian national laws. All patients signed an informed consent form in which they agreed to the use of their clinical data in an anonymous form for scientific purposes. The use of the Ligurian HIV Network database (Medinfo) for scientific purposes was approved by the Ligurian Ethics Committee (date of approval: 28 August 2013). For the purpose of the present study, all patients were sampled at one timepoint in 2016 after providing informed consent for immune cell profiling to evaluate their innate immunity (IRB approval (IRCCS AOU San Martino-IST Genova n51/09, ALS 2 n°10/2011).

HIV RNA<50 copies/ml was a requirement for inclusion into the study. All demographic, laboratory, and therapeutic data were extrapolated from the electronic database Medinfo (43) that is automatically populated by an autonomous system according to international standard communication protocols based on HTTP (HyperText Transfer Protocol) and HSSP (Healthcare Services Specification Project). The Medinfo database allowed laboratory data analysis from 2010 to the present. Viremia copy-years (VCY), the principal exposure of interest, is a time-varying measure of cumulative plasma HIV burden that was calculated between 2010 and the time of study entry (2016). Patients had mean 2.96 HIV-RNA ( ± 0.69) evaluations per year (range 1-7 HIV-RNA evaluations/year) that were all included for VCY calculation during the study period. A threshold of <50 copies/mL was used to define undetectable HIV RNA across the study period. The trapezoidal rule was used to approximate the integral representing the area under each patient’s longitudinal HIV viral load (VL) curve. VL burden for each segment (time interval between two consecutive VL values) was calculated by multiplying the mean of the two VL values by the time interval (35). The copy years/mL for each segment of a patient’s VL curve were then summed to calculate viremia copy-years. Formally, VCY is the number of copies of HIV RNA per mL of plasma over time. 10.000 copy-years of viremia is equal to having a VL of 10.000 copies/mL each day for 1 year or a VL of 1.000 copies/mL for 10 years. Virological failure was defined as confirmed HIV RNA >200 copies/mL in two subsequent blood samples or as a single HIV RNA > 1.000 copies/mL (35, 44). We defined combined ART (cART) as the administration of at least 3 antiretroviral agents including either a ritonavir-boosted protease inhibitor (PI/r), a nonnucleoside reverse transcriptase inhibitor (NNRTI), or an integrase inhibitor (INSTI).

### Sampling and Flow Cytometry

Peripheral blood (15 mL) was drawn by venipuncture and peripheral blood mononuclear cells (PBMC) were obtained by density gradient centrifugation (Ficoll-Hipaque) and cryopreserved until use. Cells were analyzed by multicolor flow cytometry(FACS Fortessa, BD, MountainView, CA, USA, as described previously (45). For analysis, cells were gated using forward and side light scatter parameters with acquisition of 10.000 events.

NK cells were defined as CD3-CD14-CD19-CD56+CD16+/-. Lin- cells were defined as CD3-CD14-CD19-. Inflammatory common lymphocyte precursors were defined by logical gates within Lin- subset as CD34+DNAM-1^bright^CXCR4+ cells and CD34-CD56-CD16+CD7- (45, 46). CD34-CD56-CD16+CD7- are recognized as a subset of CD56-CD16+ NK cells (46) previously described as “exhausted” NK cells (47).

Mean fluorescence intensity ratios (MFIr) were calculated using the formula MFI sample/MFI negative control—express mean cell molecule density. Data were analyzed using FlowJo (Tree Star, Inc.) (45, 46).

### mAbs

The following panel of mouse anti-human mAbs was used: Anti-NKp44 (Z231, IgG1), (BAT221, IgG1), anti-DNAM-1 (F22, IgG1), anti-KIR2DL2/L3/S2 (CD158b1/b2,j), anti-KIR3DL1/S1 (CD158e1/e2), anti-KIR2DL1/S1 (CD158a/h), anti-NKG2A (Z270, IgG1; Z199, IgG2a), and anti-CD85j (F278, IgG1 kindly provided by Dr. D. Pende), all of which were produced in the laboratory (A. Moretta, Genova, Italy). Anti HLA-DR (D1-12, IgG2a) was kindly provided by Dr. R. S. Accolla (University of Insubria, Varese, Italy). Commercial Goat anti-mouse were used for indirect staining in some instances and complete commercial mAb list is given in the supplementary information (**Supplementary Table 1**).

### IFN γ Production by NK Cells

PBMCs were stimulated using FcgR^+^ P815 target cells at a 10:1 E/T ratio in complete medium in the presence or absence of an anti-NKp30 and/or anti-NKp46 mAb mixture (0.1 μg/mL). Phorbol 12-myristate 13-acetate (25 ng/mL; Sigma-Aldrich, St Louis, Mo) + ionomycin (1 μg/mL; Sigma-Aldrich) was used for maximal production. GolgiPlug (BD Pharmingen) was added at 37°C from incubation start for 8 hours or after overnight incubation for 4 hours(o/n), as previously described (31). After incubation, cells were stained with mAbs followed by permeabilization/fixation (Citofix/Citoperm protocol; BD Pharmingen) and anti–IFN-γ^+^ in the presence of a permeabilizing solution. A total of 10,000 gated events were acquired.

### Quantitative HIV DNA Analysis

The procedure for measuring HIV DNA levels has been described in detail previously (20, 48).

Briefly, we reported the workflow of experiments and any changes or improvements in the procedure. Cellular DNA was isolated from a pellet of 2x10^6^ PBMCs by a DNA extraction kit following manufacturers’ instructions (QIAGEN QIAamp Blood Mini kit), following the manufacturers’ instructions. DNA amount was determined by a NanoVue Plus ND-1000 Spectrophotometer (GE Healthcare) and all purified DNAs had absorbance ratios A260/A280 of 1.7 (± 0.21). DNA recovery of 9.6 µg (± 2.5) was obtained for each sample.

Unintegrated HIV DNA (uDNA) (i.e., the ensemble of extrachromosomal viral cDNAs, including both linear cDNA and all the closed circular 1-LTR and 2-LTR and other rearranged forms) was obtained from cellular DNA by an optimized chromatographic procedure that separates high molecular weight DNA (HMW DNA) from low molecular weight DNA (LMW DNA). The uDNA was present in the eluate fraction.

Moreover the β-actin housekeeping gene was amplified in the eluate fraction and quantified on standard curve (ACTstd obtained with 10- and 2-fold serial dilutions of a reference genomic DNA (Promega) ranging from 1000 to 0.01 ng.) as control experiment on chromatographic separation demonstrating the feasibility of the procedure (49).

Total and unintegrated HIV DNA were simultaneously analyzed by a SYBR Green qPCR based method in a single run using a single set of specific primers selected in the 5’ LTR-Gag region of the HIV-1 genome, including the highly conserved primer-binding site (PBS); this method could detect all HIV-1 subtypes in the M group. PCR reactions were carried out in a 7500 real-time PCR system (Applied Biosystems, Thermo Fisher Scientific Inc) using the Hot-Rescue Real-Time PCR Kit Sybr Green (Diatheva srl). Each sample (cellular DNA or eluate fraction containing uDNA) was analyzed in six replicates, consisting of three wells containing 0.5 µg of DNA or the equivalent quantity of elution fraction and three wells containing 1.0 µg of DNA (4.5 µg, to ensure the detection of the target even in low copy numbers, i.e., near the quantification limit, QL). For samples with HIV DNA datum quantified near or detected below the QL, two additional 1 µg replicates were tested for a total of 6.5 µg of DNA (~10^6^ PBMC). In the case of negative amplification, a PCR spike test was performed by adding two or ten copies of plasmid standard to the samples to exclude the presence of inhibitors.

The HIV DNA copy number was quantified by interpolating the experimentally determined threshold cycle (CT) based on standard curves generated using half-log serial dilutions from 10^5^ to 2 copies (setting the quantification limits at 2 copies/PCR) and by adding up the copy number from the 0.5 and 1.0 µg replicates and expressed as copies/µg). The amount of integrated HIV DNA (iDNA) was obtained by subtracting the amount of uDNA from the amount of total HIV DNA (tDNA).

For the accurate enumeration of the analyzed cells in qPCR, the single copy housekeeping Rpp40 gene (part of the human RNAse P gene family) was amplified. This approach provided a control for cellular DNA degradation, presence of PCR inhibitors and input DNA normalization.

The standard curve (Rpp40std) for the quantification of a 100 bp fragment of the Rpp40 gene, (forward primer: 5’-CGTAAGCAAGTTTAGTGAATACCTGAA-3’ and the reverse primer: 5’-GCACAGCTTCCATCTTACTCAATC-3’) was made with 10- and 2-fold serial dilutions of a reference human genomic DNA (Promega) ranging from 100 to 0.01 ng [assuming 7.0 pg of DNA content per human diploid genome as conversion factor (Gregory TR. 2020. Animal Genome Size Database.

### Statistical Analysis

Data were described using mean and standard deviation (SD) for normally distributed continuous variables, median and interquartile range [IQR] for not normally distributed continuous variables, and frequency (%) for categorical and ordinal variables. A logistic regression analysis using uDNA as dependent variable was performed. All covariates with *P* < 0.25 on their univariable association with detectable uDNA were triaged for inclusion into the multivariable model. A forward stepwise variable selection approach was used in this set of covariates to retain significant variables in the final multivariable logistic regression model.

In a sensitivity analysis, a multivariable LASSO regression model was performed to ensure that only the most relevant factors associated with presence of uDNA were identified.

This penalized regression method allows for the integration of a large number of possible correlated predictors into one model and to select amongst these despite a small sample size. A general linear model using tDNA as dependent variable was performed to assess its correlations with time from birth to ART and cART initiation, VCY, and Natural Killer (NK) cell molecules expression (CD69, CD56 CD16, NKp46, and NKp30 cells)

Spearman rank correlation coefficient (*ρ*) was used to assess correlations between variables and uDNA or tDNA. Significance tests were two-sided using 0.05 significance level (JMP 9.0.1, SAS Institute).

## Results

### Study Population

**Figure 1:**
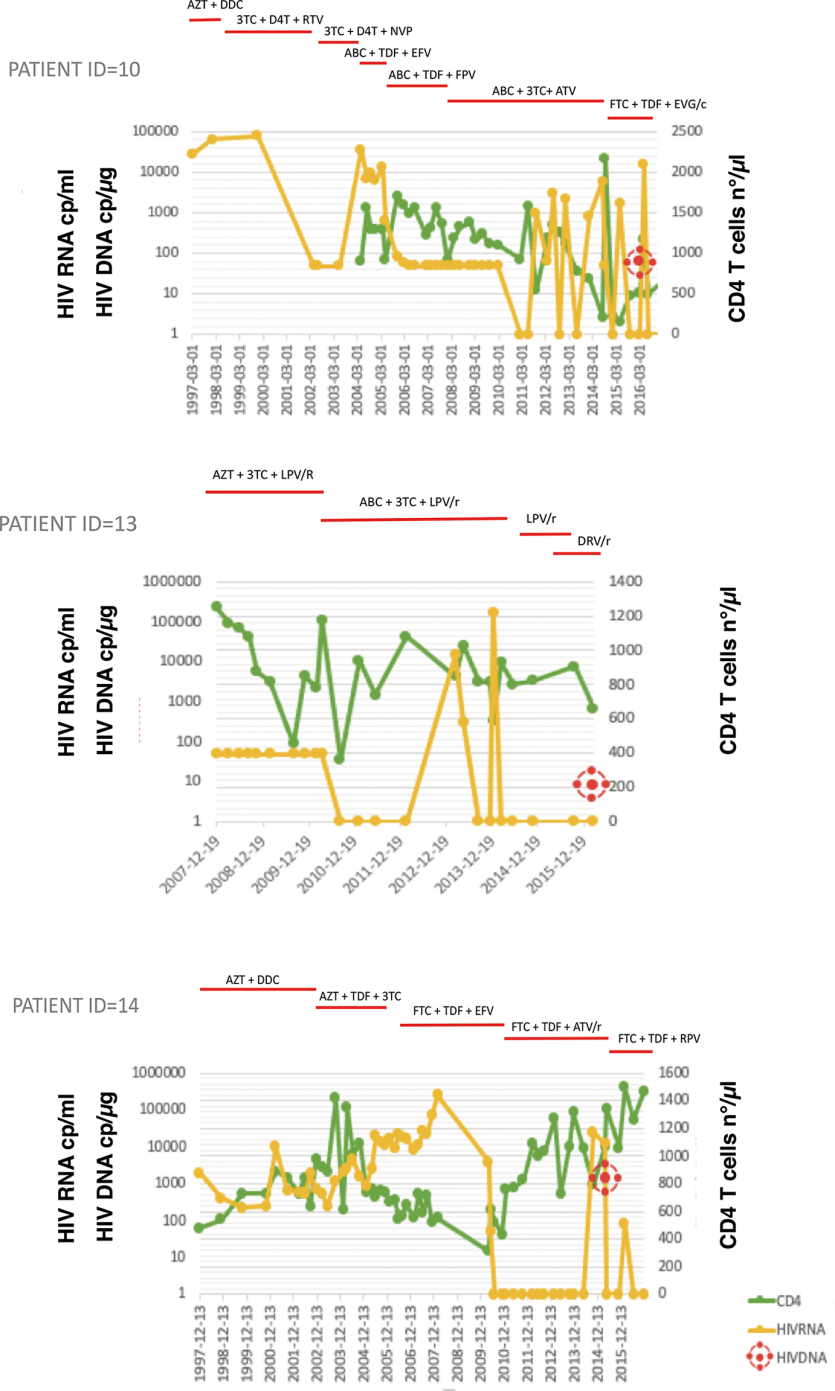
HIV-RNA and CD4+ T-cell count (CD4) trends over the years and total HIV DNA at study time in three representative patients. Treatments are shown on the top, bars indicate start and stop of relative antiretroviral medication. 3TC, lamivudine; ABC, abacavir; ATV, atazanavir; AZT, zidovudine; D4T, stavudine; DDC, zalcitabine; EFV, efavirenz; EVG/c, elvitegravir/cobicistat; FTC, emtricitabine; FPV, fosamprenavir; TDF, tenofovir disoproxil fumarate; NVP, nevirapine; RTV, ritonavir Top panel, Patient ID=10: multiple virological failures over the years due to low adherence to antiretroviral therapy (ART); Middle panel, patient ID=13: successfully treated from birth while maintaining good adherence to ART; Bottom panel, patient ID=14: treated late after birth, although with very good adherence to ART over the years.

Overall, 22 PHIV patients (41% male, 91% Caucasian) born to HIV-transmitting mothers and subsequently followed up at Ospedale Policlinico San Martino-University of Genova were enrolled in this study. The mean (SD) age at the time of analysis was 22.6 (6.1) years and all had been followed up since birth. All had been on cART for mean of 8.7 (3.9) years. Relevant clinical data are indicated in **Table 1**. Since 18 patients were born before 1996, they had been administered either monotherapy or no treatment until cART became available. Accordingly, the mean (SD) time from birth to administration of the first antiretroviral drug was 3.264 (2.719) days and time to first cART was 5.330 (2.274) days. Study participants had mean CD4+T cell count 866.5 ( ± 405.9) cells/mmc, corresponding to an average CD4 of 28.8 ( ± 15.3) % at the time of uDNA evaluation. Of the 22 PHIV, 13 (59%) were successfully treated with ART from early childhood through to adult life, 1 (5%) was treated late after birth, although with very good adherence to ART over the years, and 8 (36%), whether treated early or late, had multiple virological failures due to low adherence to ART over the years. **Figure 1** summarizes three patterns of viral replication, CD4+ cells and ART over a 9 year-period immediately before sampling for 3 representative patients. At the time of the study, shown by the red circle indicating HIV reservoir, participants had experienced different diseases courses with virological failures or viral blips over the years, as shown in **Figure 1**. Their mean VCY (from 2010 to 2016) was 20.802.6 (50.893,6) copy/years. Participants all had <50 copies/mL of HIV RNA when beginning the study. CD4+ T cell nadir was 442(236) cells/mmc, while CD4 frequency was 28.8% (15.3). **Supplementary Figure 1** gives information on all the study participants.

**Table 1:** Comparison of clinical and demographic and virological features of the study population, according to unintegrated DNA (uDNA) value, detectable vs. target not detected.

	Whole population	N	uDNA not detectable (N=10)	N	uDNA (N=10) detectable	p-value
Age, mean (SD)	23.2 (4.82)	10	22.7 (4.74)	10	23.7 (5.1)	0.94
Sex (female), n (%)	11 (55)	10	4 (40)	10	7 (70)	0.370
Caucasian ethnicity, n (%)	18 (90)	10	9 (90)	10	9 (90)	1
CD4+T cells count/mm^3^, median (range)	705 (464-2177)	10	662.5 (464-2177)	9	898 (570-1495)	**0.02**
Years of cART at study entry, median (range)	7.82 (3.79-20)	10	8.45 (3.79-16.17)	9	7.33 (4.72-20)	0.902
Days from birth to ART, mean (SD)	3576.61 (2688.93)	10	3893.7 (2451.91)	8	3180.25 (3083.28)	0.450
Days from birth to cART, mean (SD)	5568.56 (2088.67)	10	5095.7 (1635.23)	8	6159.63 (2537.34)	0.351
Viremia copy-years last 2 years (log_10_)	0.72 (2.25)	10	0.13 (1.96)	9	1.37 (2.48)	0.878
Viremia copy-years last 5 years (log_10_)	1.57 (2.43)	10	1.41 (2.31)	10	1.74 (2.65)	0.241
Viremia copy-years 2010-2016 (log_10_)	2.79 (1.43)	9	2.84 (1.15)	8	2.73 (1.77)	0.774
Total HIV DNA cp/µg DNA	16.25 (0.1-1361.46)	10	8.51 (0.1-66.99)	10	44.39 (2.65-1361.46)	0.053

### uDNA Correlates With Total HIV DNA

Total and uDNA levels were measured for all patients, iDNA was obtained by subtraction of uDNA from tDNA. A negligible cross-contamination of HMW DNA (assayed by β-actin housekeeping gene amplification) which could harbor the iDNA was found in the LMW DNA fraction with no effect on the uDNA quantification (mean 5.1% (1.3%); **Supplementary Table 2**).

**Figure 2:**
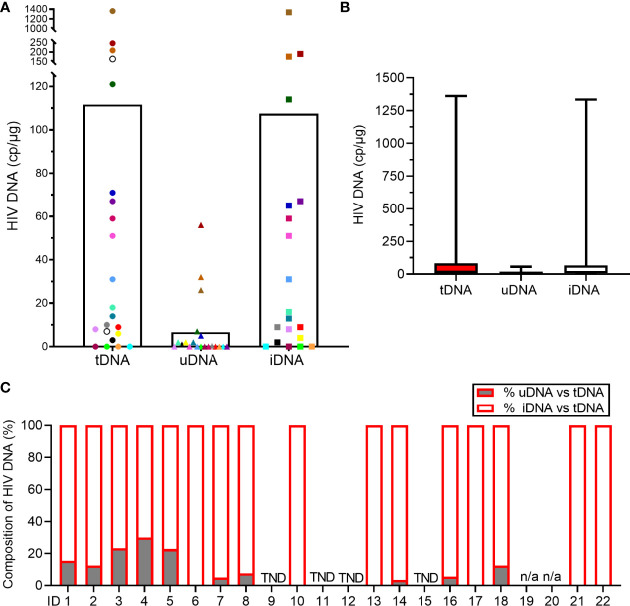
Measurements of HIV DNA reservoir in the study population. **(A)** Levels of total (tDNA), unintegrated (uDNA) and integrated (iDNA) HIV DNA. Each dot represents one patient sample and the white histogram represents the mean. Two patients’ samples did not yield sufficient cellular DNA for uDNA and iDNA analysis (open circles). **(B)** Levels of tDNA, uDNA and iDNA. Data represent the median [IQR]. **(C)** Percentages of uDNA and iDNA among tDNA in each study participant. When uDNA copy numbers were < QL of the assay, the % were reduced to 0% (ID 6, 10, 13, 17, 21, 22). When tDNA, uDNA and iDNA copy numbers were < QL, the sample is reported as TND (target not detected; ID 9, 11, 12, 15). Samples, ID 19 and ID 20, were not analyzed for uDNA and iDNA (n/a, not applicable).

Individual measurements and mean values are shown in **Figure 2A**. Median [IQR] total (tDNA), unintegrated DNA (uDNA) and integrated (iDNA) HIV DNA, were 16 [5.2-83.5], 0.25 [0-4.2] and 14.5 [2.5-66.5] copies/μg, respectively (**Figure 2B**). We evaluated the relationship of uDNA with tDNA in each patient. Unintegrated DNA levels were strongly correlated with tDNA (ρ=0.700, p=0.001) and accounted for 7.3% (9.4%) of the tDNA. Interestingly, 10 patients did not have any level of uDNA with a measurable reservoir (tDNA) in 6 cases (**Figure 2C**).

In view of individual differences in initiation of ART treatment in the cohort, we controlled by correlation analysis whether differences in time to ART initiation played a role on HIV-reservoir. A trend towards longer intervals between birth and time at the start of combined ART (time to cART) was observed in PHIV patients with higher tDNA (Beta 0.394, 95%CI -2.05;+35.84, p=0.077), while no correlation was observed with uDNA levels using Spermans’ ρ for correlations regarding uDNA(ρ=0.35, p=0.154). As expected, no association was observed between HIV reservoir and the time to any ART, including monotherapy in early years (p=0.443 for tDNA and ρ =-0.079; p=0.8567 for uDNA). These findings suggest that shades of delay in ART initiation in this cohort associate with tDNA levels and are in line with previous reports in PHIV (50, 51). Interestingly, however, there was no relationship between uDNA levels and time to ART, suggesting that factors other than ART influence presence/absence of uDNA.

Since uDNA reflects early HIV entry events in susceptible cells, and its levels were not correlated with ART-free time after birth, we asked whether HIV uDNA might associate more adequately with replicative blips as expressed by VCY in the years 2010-2016. When testing this association, neither uDNA (ρ=-0.022; p=0.9332), nor tDNA (ρ=0.1997;p=0.4269), was correlated with VCY).

Taken together, these results confirm that tDNA, as a measure of the reservoir, is correlated with time to cART, and indicate that uDNA is not correlated with time to pharmacological control of HIV replication.

### Increased uDNA Associates With NK Cell Activation and Exhaustion in cART Treated PHIV

**Figure 3:**
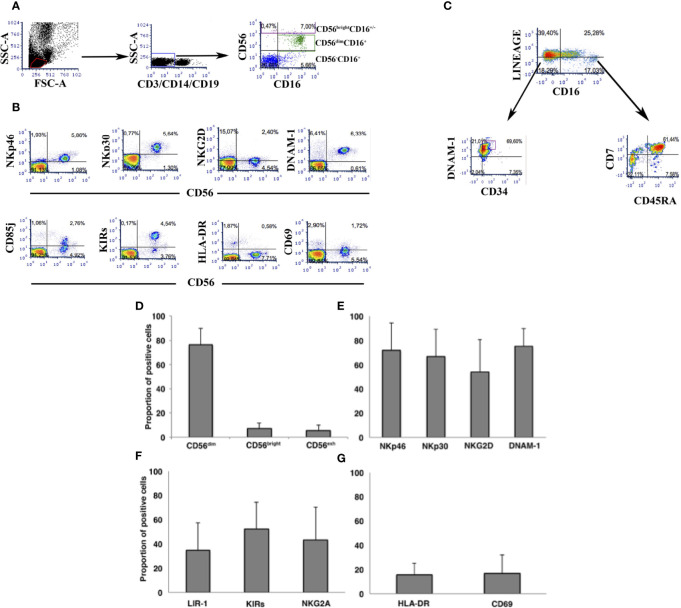
Flow cytometric analysis of peripheral NK cells in the cohort of PHIV. **(A)** Flowcytometric analysis of PBMC in a representative patient. Dot plots show gating strategy of PBMC to assess NK cell frequency. **(B)** Flowcytometric analysis of NK cells in a representative patient. CD3-14-19-56+ cells are stained for different NK cell surface antigens using directly labelled specific mAbs. **(C)** Flowcytometric analysis of a representative patient showing gating strategy and labelling to assess the frequency of (CD3-14-19-56-) and of CD34+CDNAM-1bright inflammatory cell precursors and of (CD3-14-19-56-). “Lineage” labels cells as lineage positive (CD3+14+19+56+) and lineage negative (CD3-14-19-56-)for gating and analysis of inflammatory precursors. **(D-G)** Histograms indicate mean ± SD of NK cells in PBMC of patients at the time of HIV reservoir determination. Frequencies are shown for the three major circulating NK cell subsets **(D)**, for NK cells expressing activating receptors **(E)**, inhibitory NK cell receptors **(F)**, and activation molecules **(G)**.

In view of the role played by NK cells in host immune protection and control of HIV replication contributing to HIV reservoir size, we next focused on analysis of peripheral NK cells and their possible association with the viral reservoir size and its control in PHIV patients. Flow cytometric analysis was performed on CD3-CD14-CD19-CD56- PBMC (**Figure 3A**) to study receptor expression on CD56^bright^ and CD56^dim^ NK cells (**Figure 3B**). Gating strategy to study CD34+ and CD34- common lymphocyte precursors was performed on CD3-14-19-56- PBMC (**Figure 3C**). Accordingly, analysis of the whole cohort is shown as far as main circulating NK cells (**Figure 3D**), and expression of major activating NK cell receptors (**Figure 3E**), of inhibitory receptors (**Figure 3F**) and of activation surface antigens (**Figure 3G**). Frequencies of CD56^bright^ and CD56^dim^ NK cells and those for NK cells expressing major Natural Cytotoxicity Receptors and DNAM-1 and inhibitory receptors were in line with those observed in HIV adult patients (27, 52). Persistent expression of HLA-DR and CD69 activation markers on NK cells (**Figure 3G**) is higher compared to healthy uninfected donors and is in line with original reports showing persistent NK cell activation in the presence of successful ART with VL<50 copies/mL (52, 53). Since the aim of the study was not to compare PHIV with healthy donors but rather do provide in-depth analysis of immune markers with virological data, we next assessed correlations between HIV reservoir parameters from the direct assay and Natural Killer cell frequencies or surface molecule density using Spearman’s ρ test. **Table 2** summarizes the associations observed between NK cells, circulating inflammatory precursor cells, and HIV-reservoir parameters (tDNA, uDNA).

**Table 2:** Correlation analysis of Natural Killer cell subsets with total and unintegrated HIV HNA (tDNA and uDNA).

Variable	Correlation with tDNA (Spearman’s ρ)	p-value	Correlation with uDNA (Spearman’s ρ)	p-value
**CD56^bright^CD16^+/-^**	-0,1636	0,4668	-0,1985	0,4015
**CD56^dim^CD16^+^**	-0,1236	0,5837	-0,151	0,525
**CD56^-^CD16^+^**	0,5787	0,0048	0,8248	<,0001
**CD69**	0,1761	0,4331	0,4601	0,0412
**DNAM-1**	0,2231	0,3183	-0,1231	0,6051
**CD34^+^DNAM-1^bright^CXCR4^+^**	0,2242	0,3158	0,5786	0,0075
**HLA-DR**	0,0062	0,9781	0,1585	0,5045
**KIR**	0,389	0,0736	0,02	0,9333
**LIR-1**	0,0878	0,6977	-0,0739	0,7569
**NKG2A**	0,0334	0,8827	0,2339	0,3209
**NKG2C**	0,0252	0,9113	0,0192	0,9358
**NKG2D**	-0,056	0,6954	-0.548	0.015
**NKp30**	-0,2712	0,2221	0,1354	0,5692
**NKp46**	-0,265	0,2333	-0,265	0,2333

Molecule density of CD69 on NK cells (as determined by CD69 MFIr) and CD69 frequency were not correlated with tDNA and directly correlated with uDNA (ρ=0,457, p=0.0412; ρ=0.4601, p=0.0428 respectively)The frequency of CD56^-^CD16^+^NK cells which are composed for 50% by mature NK cells and in the rest by CD34-CD56-CD16+CD7- [**Figure 3C** and (54)], were also directly correlated with uDNA levels (ρ=0.8248, p<0.0001), but not with total or integrated HIV DNA. In addition, Spearman’s analysis revealed an inverse association between the proportion of NKG2D^+^ NK cells and uDNA (ρ=-0.548, p=0.015), but not with tDNA (ρ=-0.056, p= 0.6954). In contrast, the proportion of NK cells expressing the other activating NKp46 or NKp30 molecules was not correlated with any form of HIV reservoir (**Table 2**).

### Circulating Inflammatory NK Cell Precursors (CD34+DNAM-1^bright^CXCR4+) Correlate With uDNA Viral Reservoir Forms

Following the present finding of an association between some NK cell subsets, including exhausted NK cells, and recent replication events or residual viral replication as determined by uDNA levels, we tested the hypothesis that continuous NK cell turnover may be observed in patients with higher uDNA levels. In the presence of increased peripheral turnover, there is increased bone marrow precursor activity (45, 46). To verify whether “inflammatory” NK cell precursors are released from the bone marrow in these patients, we used flow cytometry to assay the proportion of CD34^+^DNAM-1^bright^CXCR4^+^ precursors in PBMC. The proportion of these cells was directly correlated with uDNA levels (ρ=0.579, p=0.0075; Spearman’s test) thus supporting the hypothesis of increased lymphoid turnover in patients with higher uDNA levels. A direct correlation was also observed between CD34^+^DNAM-1^bright^CXCR4^+^ precursors and “exhausted” CD56^-^CD16^+^NK cells (ρ=0.520, p=0.013; Spearman’s test), thus further supporting the existence of chronic innate immune stimulation in patients with higher levels of uDNA (**Table 2**).

Univariate logistic regression included relevant associations with uDNA for CD56^-^CD16^+^, CD69MFIr, NKG2D, 2yrVCY, NKG2D, tDNA, CD34^+^DNAM^-^1^bright^CXCR4^+^ and female sex (p<0.25 in all cases). In the multivariable logistic model the frequency of CD56^-^CD16^+^, and CD34^+^DNAM-1^bright^CXCR4^+^ cell proportions, confirmed an association with uDNA levels (adjusted OR, aOR 1.61, 95%CI 1.01-2.58, p=0.045 and aOR 1.09, 95%CI 0.97-1.22, p=0.168, respectively) (**Table 3**).

**Table 3:** Association between presence of unintegrated DNA (uDNA), clinical, laboratory data and Natural Killer (NK) cell molecules expression.

	Unadjusted*		Adjusted**		Adjusted***	
	OR (95%CI)	p-value	OR (95%CI)	p-value	OR (95%CI)	p-value
Age	1.05 (0.87-1.27)	0.636				
Female Sex	3.5 (0.55-22.3)	0.185				
Ethnicity (not Caucasian vs Caucasian)	1 (0.05-18.57)	1.000				
Months from birth to ART	1 (1-1)	0.567				
Months from birth to cART	1 (1-1)	0.289				
Years of cART at study entry	1.03 (0.81-1.3)	0.832				
tDNA (cp/μg)	1.02 (0.99-1.04)	**0.154**				
VCY 2010-2016 (log_10_)	0.94 (0.47-1.88)	0.869				
VCY 5y (log_10_)	1.06 (0.73-1.54)	0.759				
VCY_2y (log_10_)	1.31 (0.84-2.03)	0.229				
CD4 (n/μl)	1 (1-1)	0.400				
CD56^bright^	0.94 (0.76-1.16)	0.553				
CD56^dim^	0.99 (0.93-1.06)	0.735				
CD56^-^CD16^+^	5.45 (0.86-34.46)	0.071	3.76 (0.26-7015.24)	0.149	1.61 (1.01-2.58)	0.045
CD34+DNAM-1^bright^CXCR4+	1.13 (1-1.27)	0.044			1.09 (0.97-1.22)	0.168
CD69%	1.02 (0.96-1.08)	0.616				
CD69 MFIr	2.8 (0.8-9.82)	0.108				
DNAM %	0.96 (0.9-1.02)	0.196				
DNAM MFIr	1.05 (0.84-1.3)	0.689				
HLA DR %	1 (0.91-1.09)	0.921				
HLA DR MFIr	0.73 (0.27-1.97)	0.537				
KIR %	1.02 (0.97-1.08)	0.488				
KIR MFIr	0.99 (0.92-1.07)	0.800				
LIR 1%	0.97 (0.92-1.03)	0.335				
LIR 1 MFIr	1.02 (0.62-1.66)	0.947				
NKG2A%	1.02 (0.96-1.09)	0.529				
NKG2A MFIr	0.93 (0.7-1.23)	0.604				
NKG2C %	0.98 (0.93-1.04)	0.491				
NKG2C MFIr	0.78 (0.5-1.23)	0.282				
NKG2D %	0.95 (0.9-1)	0.060				
NKG2D MFIr	0.88 (0.7-1.12)	0.297				
NKp30%	1.03 (0.98-1.08)	0.232				
NKp30 MFIr	1.02 (0.91-1.15)	0.706				
NKp46%	1.01 (0.97-1.05)	0.623				
NKp46 MFIr	0.76 (0.46-1.23)	0.262				

%: frequency; 95%CI: 95% Confidence intervals; aOR, adjusted Odds Ratio; ART, antiretroviral therapy; cART, combined antiretroviral therapy; MFIr, mean fluorescence intensity ratio (molecule density) OR, Odds Ratio; tDNA, total DNA; uDNA unintegrated DNA; VCY viremia copy years [in the period 2010-2016; in last 5 years (VCY 5y); in the last 2 years (VCY 2y)].

Bold font indicates p values <0.25.

*estimated from univariable logistic regression.

**estimated from multivariable logistic regression, adjusting for all the variable with a p<0.25 at the univariate analysis (Female sex, tDNA, CD3+, CD56^-^CD16^+^, CD34+DNAM-1^bright^CXCR4+, CD69 MFIr, DNAM %, NKG2D %).

***Odds ratio and their 95%CI were obtained from an ordinary multivariable logistic regression model using the variables selected by the LASSO logistic regression. The variables included in the LASSO regression and their corresponding coefficients were CD56^-^CD16^+^ (ß=0.059) and CD34+DNAM-1^bright^CXCR4+ (ß=0.003).

**Figure 4:**
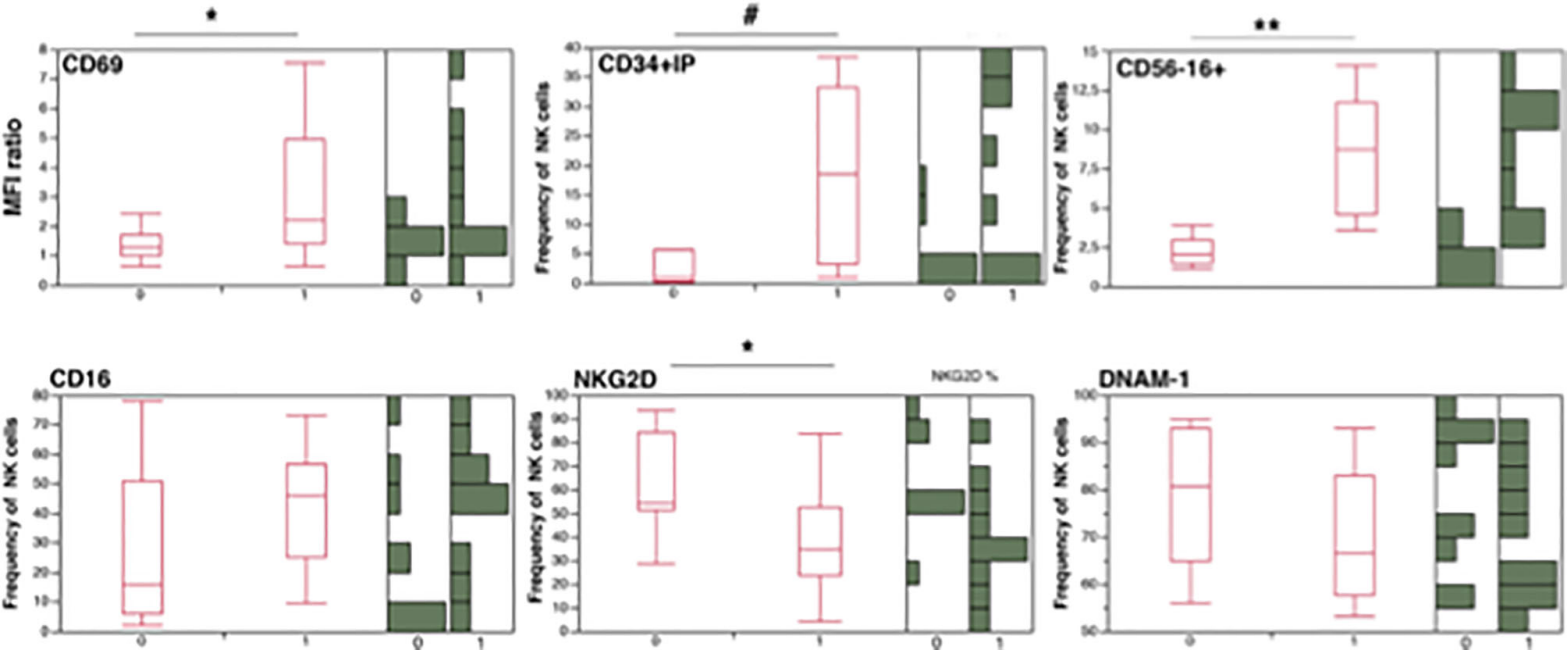
Natural killer and inflammatory precursor cell frequencies in PHIV patients after stratification for presence (1 = uDNA+) or absence (0 =uDNA-) of detectable uDNA. Lateral histograms (Green) show individual measurement frequencies *p<0.05; **p<0.01; #p<0.001; CD34+IP= CD34^+^ inflammatory precursors (CD34^+^DNAM-1^bright^CXCR4^+^).

To provide further visual consistency to correlation analysis, we also show stratified patients according to presence (uDNA+) or absence (uDNA-) of linear and circular forms of uDNA in their CD4+ PBMC in **Figure 4**. Patients with detectable uDNA had significantly increased CD69 expression, increased circulation of inflammatory CD34+ precursors and of “exhausted”CD56^-^16^+^ cells (including CD34- inflammatory precursors) and had lower frequencies of NK cells expressing the activating NKG2D receptor.

**Figure 5:**
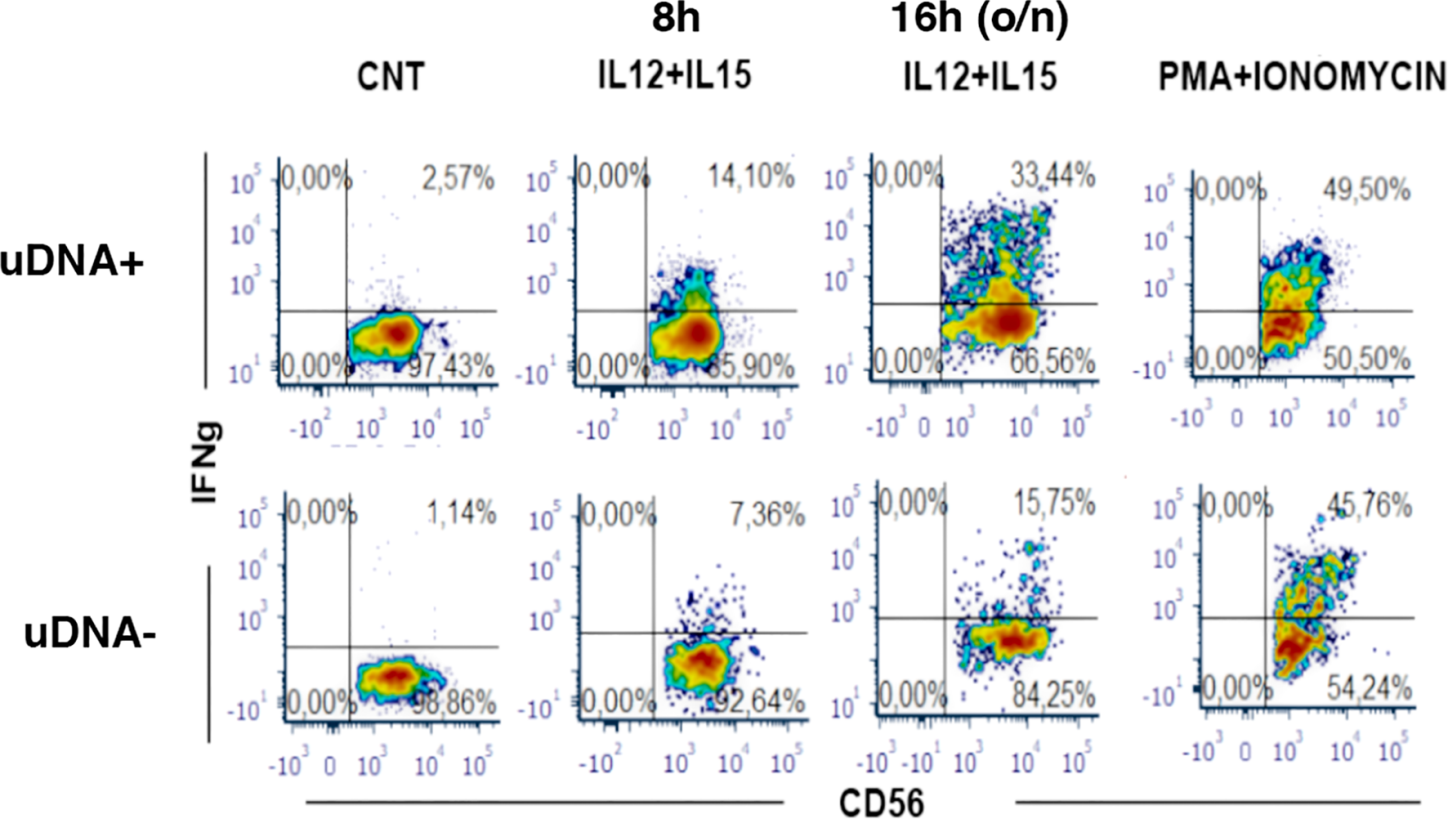
IFN-γ production is conserved in peripheral blood Natural Killer cells from cART-treated and plasma HIV-RNA suppressed patients with presence of uDNA. Flow cytometric analysis of IFN-γ production by CD56+ NK cells upon 8h- and 16h-stimulation *in vitro* with IL-12+IL-15. Medium alone as negative control (CNT) or maximal stimulus as positive control (PMA+IONO) are also shown. Upper row: uDNA positive patient (uDNA +), lower row: uDNA negative patient (uDNA -).

These observations raised a relevant issue on whether increased NK cell activation and turnover in uDNA+ virologically suppressed cART-treated patients would be due to defective NK cell function, or rather whether it could reflect increased requirements of NK cell surveillance with conserved NK cell activity thus leading to increased peripheral turnover. To address this question, we therefore studied NK cell function in uDNA+ and uDNA- patients upon cytokine stimulation. NK cell exposure to rIL-12 and rIL-15 was used to reproduce *in vitro* NK cell interaction with mature DCs. As shown in **Figure 5**, in a representative uDNA+ patient, the specific IFNγ production (see methods) was 24.6 and 65.8% after 8 and 16 hours (o/n) stimulation while in uDNA- patients NK cell IFNγ production was 14% and 32.8%. Thus, we detected intact and possibly increased IFNγ production upon rIL-12+rIL-15 stimulation in patients with residual replication as shown by uDNA detection in viral reservoir.

These data therefore indicate that in virologically suppressed patients with residual replication (uDNA+) NK cells are functional and are continuously recruited as shown by the increase and correlation with “inflammatory” precursors and signal increased peripheral turnover.

This increased NK cell turnover with conserved NK cell function may be poised to control residual replication events escaping cART.

## Discussion

In the present work, we observed direct correlations between uDNA levels (which include both linear and circular HIV DNA) and tDNA, and overall activation and turnover of NK cells.

Patients in this cohort were infected perinatally at times with no or little access to immediate or early ART, which was unavailable at the time. Accordingly, they may be representative of PHIV patients currently born in areas where access to ART is still difficult (55). In PHIV, the HIV DNA reservoir set point is rapidly established after acute HIV Infection (56), which corresponds to neonatal age in vertically infected patients, and builds up through all stages of physical and immunological maturation. Early ART induces a sizeable containment of intact proviral sequences and affects adaptive and innate immune parameters (30), while delayed treatment initiation leads to the persistence of intact provirus for at least 7-9 years (57). Accordingly, reduced tDNA levels have been associated with younger age at ART initiation in PHIV patients (58), while higher levels are considered predictive of HIV disease progression (59).

We here observed a direct correlation between uDNA and tDNA, in line with previous reports (60, 61). The transcription and translation of uDNA prior to integration is thought to aid productive viral infection and to result in either preintegration latency or productive infection, and might reflect recent HIV entry in a cell and a viral reservoir, at least in some circumstances (62). Accordingly, these data suggest that uDNA could be used, even in patients with undetectable plasma HIV RNA, to estimate a recently established component of the HIV reservoir.

The present observation that uDNA—despite its lability—provided a reliable estimate of viral burden could be explained by higher levels of total reservoir (tDNA) in PHIV patients with ongoing viral replication that is below the threshold of detectability (25, 26). This point is supported by the observation that uDNA levels were directly associated with tDNA and only had a trend towards association with VCY over shorter periods of observation, thus suggesting that uDNA may reflect recent replication and preintegration events, in line with previous reports (63). VCY directly correlates with higher mortality and organ damage in people living with HIV, and is considered a reliable marker of viral burden over years (35, 36). Since its estimation is unfeasible in patients with incomplete past virological history, uDNA levels could be a more readily available useful surrogate marker for VCY, thus integrating in a single sampling point information on recent virological history of any patient. Accordingly, future work could usefully validate uDNA assays to provide a proxy for ongoing transient blips in replication in larger populations.

In view of the known role of NK cells in HIV-1 reservoir containment (26, 27, 29) and the positive effects of the successful control of viral replication on NK cell phenotypic landscape (30), we studied the relationship between DNA forms (uDNA, tDNA, iDNA) and NK cell phenotype and turnover by analyzing CD34^+^DNAM-1^bright^CXCR4^+^ inflammatory common lymphocyte precursors circulating in PBMC (45, 46). PHIV patients with higher uDNA levels had higher NK cell turnover, as shown by increased exhausted NK cells (CD56-CD16+) and increased CD34^+^DNAM-1^bright^CXCR4^+^ inflammatory precursors. Moreover, the expression of CD69, a marker of NK activation (27), was directly correlated with uDNA in PBMC, indicating higher activation in patients with recent replication events. Taken together, these data are in line with the known direct role of innate immunity in controlling HIV replication. In this context, the presence of residual HIV replication, as expressed by detectable uDNA levels, does not represent a lack of ART activity alone since all patients were successfully on treatment and had undetectable HIV RNA (<50 cp/ml) at the time of testing, but only some of them had undetectable uDNA. Accordingly, persistence of uDNA in only a fraction of them accompanied to NK cell parameters of activation and by increased output of “emergency” precursors to mature NK cells may be interpreted as an imbalance in immune control of HIV in these patients. This is supported by the notion that NK cell function and phenotype contribute to controlling both HIV replication (21, 28–30) and the HIV DNA reservoir (22). At the same time, the high frequency of NK CD34^+^DNAM-1^bright^CXCR4^+^ precursors in the peripheral blood of patients with higher proportions of exhausted CD56^-^CD16^+^NK cells could be interpreted as an attempt to rapidly reconstitute mature NK cells undergoing peripheral turnover in patients with detectable uDNA and incomplete immunological control (46). In addition, clonal expansion directed towards diverse antigens including CMV, flu, EBV rather than passive expansion could represent an important aspect in reservoir maintenance. Depending on efficiency of translocation, viral replication, innate immune mechanisms and CD8+CTL function, variable CD4 clonal expansion could be envisaged, with different levels of involvement of NK cells to control the renovating reservoir (64).

Measures of intact proviral copies provide useful information for cure-oriented attempts towards functional cure and do not provide data on recent replication events (12, 65, 66). In the present work, uDNA provides direct information on HIV reservoir dynamic status and correlates with residual “immune-fatigue” and peripheral turnover as shown by the present data on inflammatory precursor and NK cell phenotype/function. This may be regarded as information complementary to other HIV reservoir assays that may become clinically useful to support and monitor functional cure trials.

Some limitations apply to the present analysis. We had no control over ART adherence, possibly confounding study results. In addition, we evaluated HIV DNA in a single sample and could not assess its trend over time over repeated measures, which will be needed in future to transfer this knowledge into clinical usefulness. However, the study has the strengths of evaluating, in a very specific population of vertically infected youths, a new way to estimate recent HIV reservoir generation that cannot be evaluated by other assays and linking reservoir clearance with a study of innate immune response.

In conclusion, in aviremic PHIV patients even after prolonged suppression of viral replication on ART, HIV reservoir displays signs of turnover. Detection of HIV-1 uDNA associates with evidences of NK cell intervention and peripheral turnover without functional impairment. Measures of turnover in HIV reservoir and in immune responses with uDNA and inflammatory precursor assays may offer additional insight to targeted personalized choices for functional HIV eradication.

## Data Availability Statement

The raw data supporting the conclusions of this article will be made available by the authors, without undue reservation.

## Ethics Statement

The studies involving human participants were reviewed and approved by Ligurian Ethics Committee. The patients/participants provided their written informed consent to participate in this study.

## Author Contributions

LT: Data Curation, Investigation, Formal analysis, Validation, Visualization, writing. FeB: Data Curation, Investigation, Formal analysis, Validation, Visualization. AC Investigation, Formal analysis, Validation, Writing. CO: Data Curation, Investigation, Formal analysis, Validation. FrB: Statistical analysis, Validation. SM: Formal analysis, Validation. MG: Formal analysis, Validation. LM: Investigation, Funding acquisition, Writing. MM: Validation, Writing. AB: Conceptualization, Supervision, Data curation, Validation. AM: Conceptualization, Formal analysis, Funding acquisition, Validation, Writing, Supervision. All authors contributed to the article and approved the submitted version.

## Funding

This work was supported by grants awarded by 5x1000 - Immunity in Cancer Spreading and Metastasis (ISM) Project Code: 21147; IG 5x1000 Molecular Clinical Oncology Extension Program - Project Code: 9962; AIRC INVESTIGATOR GRANT (LM) IG 2017 - Project Code: 19920; IG 2014 - Project Code: 15283; Istituto Superiore di Sanita` (I.S.S.): Programma nazionale di ricerca sull’AIDS, Accordi di collaborazione scientifica 45G.11 (A.D.M.), 40H69 (A.D.M.); MIUR: FISR2020IP_02937 (ADM).

## Conflict of Interest

The authors declare that the research was conducted in the absence of any commercial or financial relationships that could be construed as a potential conflict of interest.

## Publisher’s Note

All claims expressed in this article are solely those of the authors and do not necessarily represent those of their affiliated organizations, or those of the publisher, the editors and the reviewers. Any product that may be evaluated in this article, or claim that may be made by its manufacturer, is not guaranteed or endorsed by the publisher.

## Supplementary Material

Supplementary Figure 1 Description of replication and immune parameters in the patient cohort. One panel for each patient. Each panel represents HIV RNA (cp/mL), HIV DNA (cp/10^6^ PBMC), and CD4 T cell counts (cells/mmc) from 2000 to 2016 in the 22 PHIV enrolled in the study. Triangles indicate uDNA levels at the time of the assay in representative patients.
