# Supplementary figures and images for: Persistence of Unintegrated HIV DNA Associates With Ongoing NK Cell Activation and CD34+DNAM-1brightCXCR4+ Precursor Turnover in Vertically Infected Patients Despite Successful Antiretroviral Treatment
Source: Front Immunol. 2022 Apr 26;13:847816. doi: 10.3389/fimmu.2022.847816 (PMC9088003; doi:10.3389/fimmu.2022.847816)

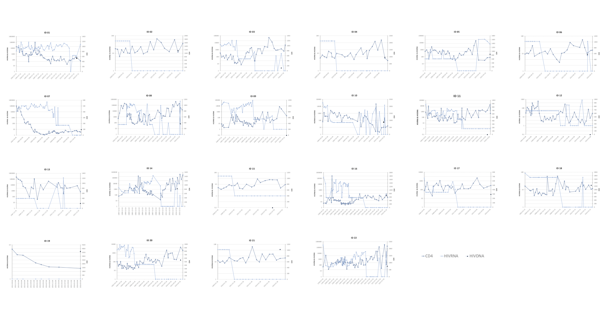

Supplement: Supplementary Figure 1 — Description of replication and immune parameters in the patient cohort. One panel for each patient. Each panel represents HIV RNA (cp/mL), HIV DNA (cp/106 PBMC), and CD4 T cell counts (cells/mmc) from 2000 to 2016 in the 22 PHIV enrolled in the study. Triangles indicate uDNA levels at the time of the assay in representative patients. [file Image_1.tiff]
