# Supplementary material for: Persistence of Unintegrated HIV DNA Associates With Ongoing NK Cell Activation and CD34+DNAM-1brightCXCR4+ Precursor Turnover in Vertically Infected Patients Despite Successful Antiretroviral Treatment
Source: Front Immunol. 2022 Apr 26;13:847816. doi: 10.3389/fimmu.2022.847816 (PMC9088003; doi:10.3389/fimmu.2022.847816)
Supplement: Supplementary file 2 [file Table_1.docx]

**Supplementary Table 1**

| **REAGENT or RESOURCE** | **SOURCE** | **IDENTIFIER** |
| --- | --- | --- |
| **Antibodies** | | |
| CD56 PeCy7 | Immunotech-Coulter, Marseille, France | Item N° A21692 |
| CD34 PeCy7 | BD Pharmigen, San Jose, CA, USA | CAT 560710 |
| CD34 FITC | BD Pharmigen, San Jose, CA, USA | CAT 555821 |
| CD38 PerCp/Cy5.5 | BioLegend, San Diego, CA, USA | CAT 303522 |
| CD3 APC: | BioLegend, San Diego, CA, USA | CAT 300312 |
| CD3 FITC | San Diego, CA, USA | CAT 300306 |
| CD3 BV510 | BD Pharmigen, San Jose, CA, USA | CAT 563109 |
| CD14 BV510 | BD Pharmigen, San Jose, CA, USA | CAT561391 |
| CD19 BV510 | BD Pharmigen, San Jose, CA, USA | CAT 562947 |
| Lineage Cocktail 2 (lin2) (CD3, CD14, CD19, CD20, CD56) FITC | BD Pharmigen, San Jose, CA, USA | CAT 643397 |
| CD107a PE | BD Pharmigen, San Jose, CA, USA | CAT 555801 |
| CD16 APC-CY7 | BD Pharmigen, San Jose, CA, USA | CAT 557758 |
| CXCR4 Purified | BD Pharmigen, San Jose, CA, USA | CAT 555972 |
| Perforin PE | BioLegend, San Diego, CA, USA | CAT 308106 |
| DNAM-1 APC | BioLegend, San Diego, CA, USA | CAT 338312 |
| NKG2D PerCp/Cy5.5 | BioLegend, San Diego, CA | CAT 320818 |
| NKp30 Alexa Fuor® 647 | BD Pharmigen, San Jose, CA, USA | CAT 558408 |
| NKP46 HorizonV450 | BD Pharmigen, San Jose, CA, USA | CAT 562099 |
| HLADR PerCp/Cy5.5 | BD Pharmigen, San Jose, CA, USA | CAT 560652 |
| CD69 PE | BioLegend, San Diego, CA, USA | CAT 310906 |
| NKG2C Purified | R&DSystems, Inc. Minneapolis, MN 55413 | CAT MAB1381 |
| Goat Anti-Mouse IgG1 PE | Beckman-Coulter, Marseille, France | CAT 731840 |
| Goat Anti-Mouse IgG2b FITC | Southern Biotech, Birmingham, AL, USA | CAT 1090-02 |
| Goat Anti-Mouse IgG2a FITC | Southern Biotech, Birmingham, AL, USA | CAT1080-02 |
