# Supplementary material for: Persistence of Unintegrated HIV DNA Associates With Ongoing NK Cell Activation and CD34+DNAM-1brightCXCR4+ Precursor Turnover in Vertically Infected Patients Despite Successful Antiretroviral Treatment
Source: Front Immunol. 2022 Apr 26;13:847816. doi: 10.3389/fimmu.2022.847816 (PMC9088003; doi:10.3389/fimmu.2022.847816)
Supplement: Supplementary file 3 [file Table_2.docx]

**Supplementary Table 2** Cross-contamination level of high molecular weight DNA (HMW DNA) measured in eluate fraction by qPCR of β-actin housekeeping gene

| **Sample** | **ng of gDNA/qPCR (2 µl)** | **ng of gDNA/µl of eluate fraction** | **Total volume of eluate fraction (µl)** | **ng of gDNA in eluate fraction** | **Starting amount of gDNA loaded in the column (ng)** | **Contamination of LMW DNA by HMW DNA (%)** |
| --- | --- | --- | --- | --- | --- | --- |
| ID_1 | 3,1 | 1,6 | 110 | 170,5 | 3529 | 4,8% |
| ID_2 | 1,9 | 1,0 | 106 | 100,7 | 3529 | 2,9% |
| ID_3 | 3,5 | 1,8 | 109 | 190,75 | 3529 | 5,4% |
| ID_4 | 2,8 | 1,4 | 103 | 144,2 | 3529 | 4,1% |
| ID_5 | 3,2 | 1,6 | 106 | 169,6 | 3529 | 4,8% |
| ID_6 | 2,6 | 1,3 | 102 | 132,6 | 3529 | 3,8% |
| ID_7 | 4,7 | 2,4 | 105 | 246,75 | 3529 | 7,0% |
| ID_8 | 3,0 | 1,5 | 109 | 163,5 | 3529 | 4,6% |
| ID_9 | 2,7 | 1,4 | 103 | 139,05 | 3529 | 3,9% |
| ID_10 | 2,5 | 1,3 | 105 | 131,25 | 3529 | 3,7% |
| ID_11 | 3,3 | 1,7 | 105 | 173,25 | 3529 | 4,9% |
| ID_12 | 4,2 | 2,1 | 106 | 222,6 | 3529 | 6,3% |
| ID_13 | 2,4 | 1,2 | 107 | 128,4 | 3529 | 3,6% |
| ID_14 | 4,5 | 2,3 | 108 | 243 | 3529 | 6,9% |
| ID_15 | 4,6 | 2,3 | 108 | 248,4 | 3529 | 7,0% |
| ID_16 | 2,6 | 1,3 | 109 | 141,7 | 3529 | 4,0% |
| ID_17 | 3,5 | 1,8 | 109 | 190,75 | 3529 | 5,4% |
| ID_18 | 4,0 | 2,0 | 110 | 220 | 3529 | 6,2% |
| ID_19 | n/a | n/a | n/a | n/a | n/a | n/a |
| ID_20 | n/a | n/a | n/a | n/a | n/a | n/a |
| ID_21 | 3,6 | 1,8 | 104 | 187,2 | 3529 | 5,3% |
| ID_22 | 2,8 | 1,4 | 103 | 144,2 | 3529 | 4,1% |
| **Mean** | **3,3** | **1,6** | **106** | **174,4** | **3529** | **4,9%** |
| **SD** | **0,8** | **0,4** | **2** | **43,5** | **0** | **1,2%** |

The contamination from HMW DNA fragments was evaluated in PCR reaction (ng/qPCR, 2 µl of eluate fraction were tested in triplicate) and in the total volume of the eluate fraction (varying from sample to sample). Percentage of contamination is expressed as % versus the starting amount of cellular DNA (gDNA) loaded in the chromatographic column (3529 ng, ref.49).
